# Alternative splicing regulates distinct subcellular localization of Epithelial splicing regulatory protein 1 (Esrp1) isoforms

**DOI:** 10.1038/s41598-017-03180-3

**Published:** 2017-06-20

**Authors:** Yueqin Yang, Russ P. Carstens

**Affiliations:** 1 Department of Genetics, Perelman School of Medicine, University of Pennsylvania, Philadelphia, Pennsylvania 19104 USA; 2 Department of Medicine, Perelman School of Medicine, University of Pennsylvania, Philadelphia, Pennsylvania 19104 USA

## Abstract

Epithelial-Splicing-Regulatory-Protein 1 (Esrp1) is a cell-type specific RNA-binding protein (RBP) that is essential for mammalian development through maintenance of epithelial cell properties including barrier function. Esrp1 also regulates splicing during the epithelial to mesenchymal transition (EMT). It contains three highly conserved RNA recognition motifs (RRMs) in the absence of other clearly defined protein domains. *Esrp1* itself is also alternatively spliced to produce multiple protein isoforms. Here we determined that two competing alternative 5′ splice sites in exon 12 yield Esrp1 isoforms with differential nucleocytoplasmic localization. We carried out a detailed characterization of the Esrp1 peptide that is sufficient to confer nuclear localization. Furthermore, we identified splice variants encoding distinct nuclear and cytoplasmic isoforms of fusilli, the *D*. *Melanogaster* Esrp1 ortholog. Our observations demonstrate that the production of both nuclear and cytoplasmic Esrp1 isoforms through alternative splicing is phylogenetically conserved; strongly suggesting it is biologically significant. Thus, while previous studies have described extensive regulation by nuclear Esrp1 to promote epithelial specific splicing, it will be of great interest to study the contribution of cytoplasmic Esrp1 in maintenance of epithelial cell functions.

## Introduction

RNA binding proteins (RBPs) play crucial roles at each step in RNA processing, including 5′ capping, splicing, polyadenylation, mRNA transport, localization, mRNA stability, and translation. Emerging evidence suggests that many RBPs regulate multiple steps in RNA processing^1–4^. For example, the embryonic lethal abnormal vision like 1 (ELAVL1, also known as HuR) can regulate alternative splicing and promote mRNA stability and/or translation efficiency^2, 5^. In some cases, the multifunctional roles of RBPs depend on the ability of a single gene to produce multiple isoforms with different nucleocytoplasmic localization through alternative splicing. For example, three splice isoforms of QKI with differential subcellular localization have been found to regulate multiple post-transcriptional processes, including splicing, mRNA localization, mRNA stability and protein translation^6–9^. Recent studies have also revealed novel functions of the well-known splicing factor Rbfox1 in regulation of mRNA stability and translation through the production of cytoplasmic isoforms by alternative splicing^10^. Hence, it is increasingly recognized that many RBPs that have well characterized roles in one specific step of post-transcriptional regulation may have other unrecognized functions in RNA processing. Emerging evidence suggests that integrated post-transcriptional regulation by a specific RBP can function to shape biologically coherent post-transcriptional regulons^1^.

Our lab previously identified Epithelial Splicing Regulatory Proteins 1 and 2 (ESRP1 and ESRP2) as epithelial cell-type-specific proteins that enforce genome-wide epithelial splicing programs in diverse epithelial cell types^11–13^. Loss of ESRP1 and ESRP2 expression during the Epithelial to Mesenchymal Transition (EMT) plays a major role in splicing switches that occur during this crucial developmental process that also has disease relevant consequences^11, 13, 14^. Taken together, our previous studies demonstrated that the ESRPs regulate alternative splicing of hundreds of gene transcripts. Our group developed *Esrp1* knockout (KO) mice and showed that they were post-natal lethal with fully penetrant cleft lip associated with cleft palate (CL/P)^15^. Whereas *Esrp2* KO mice had no apparent defects, *Esrp1*/*Esrp2* double knockout mice manifest more extensive developmental phenotypes than *Esrp1* KO mice, including abnormal upper limb development, lung and salivary gland agenesis, colonic atresia, and epidermal defects^15^. Hence, while there is some redundancy in the function for Esrp1 and Esrp2, both *in vitro* and *in vivo* evidence indicate that Esrp1 has a more crucial role. Furthermore, conditional ablation of Esrp1 and Esrp2 in the skin resulted in a lethal epidermal barrier defect indicating that the Esrps are also required for maintenance of epithelial cell barriers^15^.

Esrp1 is a highly conserved RBP, with orthologs in all vertebrates as well as *D*. *Melanogaster* (fusilli) and *C*. *elegans* (sym-2) that are also regulators of alternative splicing^12, 16^. It has three highly conserved RNA recognition motifs (RRMs) in the absence of other clearly defined protein domains. Several alternative splicing events that are conserved between human and mouse are present in the C-terminus of Esrp1, downstream of all three RRM domains, including an alternative 5′ splice site (5′ss) at the end of exon 12 and two consecutive cassette exons (exon 14 and 15) that can be included individually, together in tandem, or skipped. While our previous studies have demonstrated a role for Esrp1 as a splicing factor in the nucleus, a recent study described a function of Esrp1 in the cytoplasm to regulate translation^17^. Based upon examples of other splicing factors with nuclear and cytoplasmic isoforms generated through alternative splicing, we considered the possibility that different Esrp1 splice isoforms might account for this differential localization. In this study, we demonstrate that differential subcellular localization of Esrp1 results from the alternative 5′ splice sites at the end of exon 12. We identified the minimal peptide sequence that is necessary and sufficient for nuclear localization of Esrp1 nuclear isoforms, which we propose is most likely to function as a nuclear localization signal (NLS) and is therefore designated a putative NLS (pNLS). This element is different from previously characterized NLS consensus sequences. We also determined the key residues in the Esrp1 pNLS. We further showed that the production of both nuclear and cytoplasmic isoforms through alternative splicing is also exhibited by the fly ortholog fusilli. These findings strongly suggest that Esrp1 and its orthologs have conserved functions in yet-to-defined post-transcriptional regulatory roles in the cytoplasm beyond splicing regulation in the nucleus.

## Results

### Choice of different alternative 5′ splice sites downstream of exon 12 gives rise to Esrp1 isoforms with differential subcellular localization

**Figure 1:**
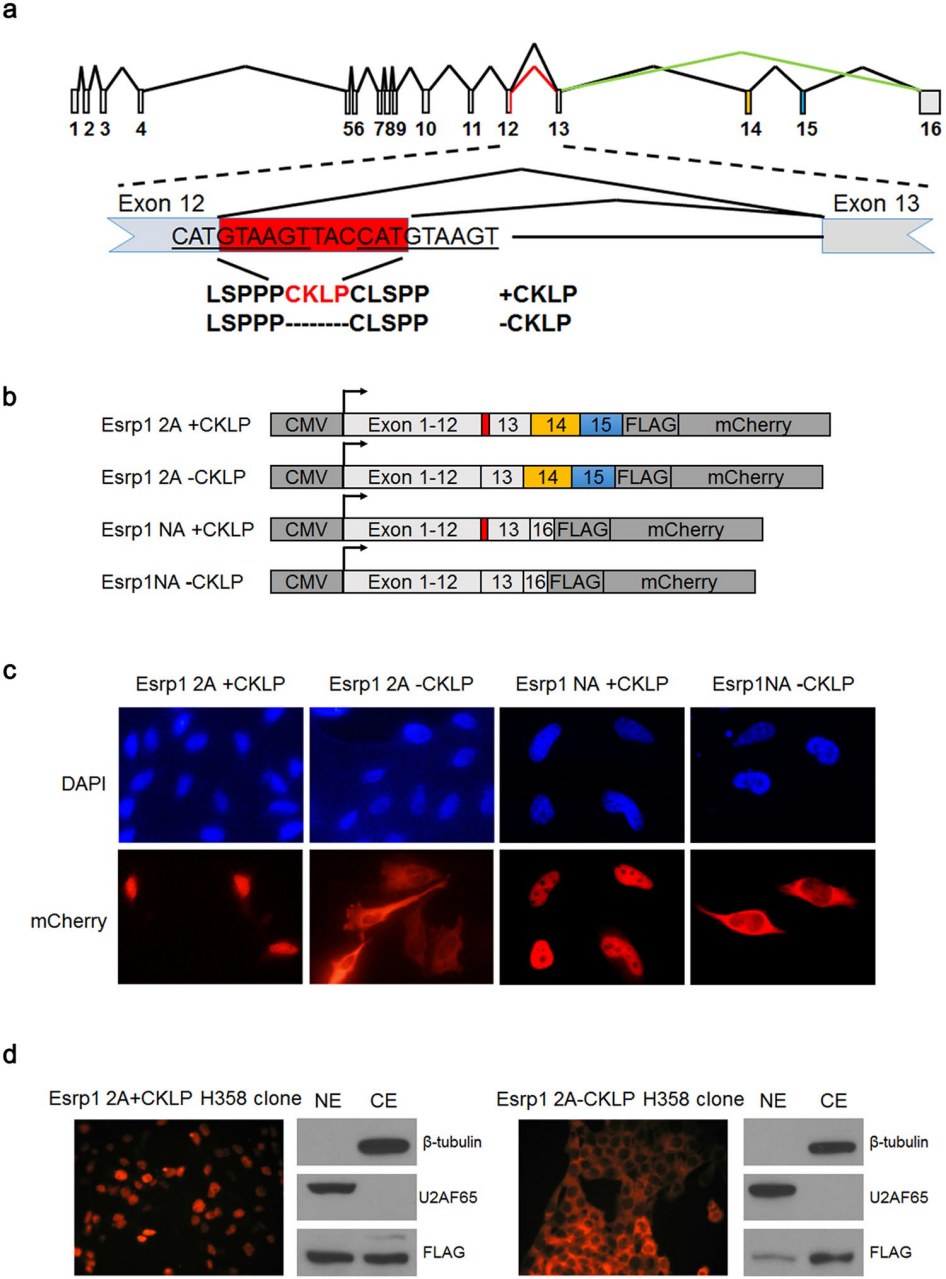
Choice of different alternative 5′ splice sites downstream of exon 12 gives rise to Esrp1 isoforms with differential subcellular localization. (**a**) Schematic of the genomic organization and alternative splicing of *Esrp1*. Gray boxes indicate constitutive exons. Exons 14 (yellow box) and 15 (blue box) are cassette exons, and exon 12 has an alternative 5′ splice site (highlighted in red) with the sequences shown below. (**b**) Schematic of vectors expressing cDNAs for the different full length Esrp1 isoforms as FLAG and mCherry fusion proteins for transfection and microscopy in (**c**). (**c**) Representative images of HeLa cells (20x) transfected with expression vectors of the different Esrp1 isoforms depicted in (**b**), showing that CLKP is required for predominant nuclear localization. mCherry represents the localization of Esrp1 protein, and DAPI represents the nucleus. For each transfection, a qualitative measurement of subcellular localization was quantified for at least 20 cells from at least 3 randomly chosen microscopic fields as described in Methods. The same measurements were done for each transfection in subsequent experiments and these quantifications are summarized in Table 1. (**d**) mCherry fluorescence and Western blots of nuclear and cytoplasmic fractions from human H358 stable clones expressing full length Esrp1 2A + CKLP or 2A − CKLP isoforms confirmed predominant nuclear localization for Esrp1 2A + CKLP protein isoform and cytoplasmic localization for Esrp1 2A − CKLP protein isoform. β-tubulin was used as a cytoplasmic marker, and U2AF65 was used as a nuclear marker.

The alternative 5′ splice sites in *Esrp1* exon 12 consists of two identical 5′ splice site consensus sequences that are separated by 12 nucleotides (5-TGAAGTTACCAT-3), usage of which generates protein isoforms that differ by four amino acids “Cys-Lys-Leu-Pro” (CKLP) (Fig. 1a). Cassette exons 14 and 15, which are 151 nt and 72 nt in length respectively, can be included or skipped individually or together. While translation terminates in exon 16 when both exons are skipped (NA), inclusion of both exons (2A) introduces a stop codon in exon 15 (Fig. 1a,b). To investigate whether the sequence required for nuclear localization is encoded by the differential splicing of alternative 5′ splice sites downstream of exon 12 or exons 14 and/or 15, we generated cDNA constructs expressing four full length mouse Esrp1 protein isoforms (2A + CKLP, 2A − CKLP, NA + CKLP and NA − CKLP) fused with a fluorescence tag (mCherry) and transfected them into HeLa cells (Fig. 1b). As shown in Fig. 1c, isoforms containing the CKLP amino acids (derived from the distal 5′ss) were predominantly nuclear while isoforms lacking the CKLP (derived from the proximal 5′ss) were predominantly localized to the cytoplasm. However, the inclusion or skipping of exon 14 and 15 together did not influence subcellular localization (Fig. 1c). Quantification of cells with different subcellular localization patterns from each transfection in this and subsequent experiments are summarized in Table 1. We also did not detect differences in localization of Esrp1 isoforms that resulted from inclusion or skipping of exons 14 and 15 alone (data not shown). These observations suggested that CKLP is part of an NLS required for the nuclear localization of Esrp1. To further support the presence of different isoforms of Esrp1 in the nucleus and cytoplasm, we constructed stable clones that express either the nuclear (2A + CKLP) or cytoplasmic (2A-CKLP) isoform as a mCherry fusion protein in human H358 epithelial cell line. As shown in Fig. 1d, mCherry fluorescence is primarily detected in the nucleus for Esrp1 2A + CKLP clone and predominantly in the cytoplasm for Esrp1 2A-CKLP clone. Furthermore, we obtained cytoplasmic and nuclear fractions from both H358 stable clones through subcellular fractionation (Fig. 1d). For the Esrp1 2A-CKLP protein, we noted a greater amount of Esrp1 in the cytoplasm compared to the Esrp1 2A + CKLP protein. For the Esrp1 2A + CKLP clone, we detected Esrp1 protein in both the nucleus and cytoplasm. While the reason we detect the protein in the cytoplasmic fraction is not clear, it may be related to leakage of soluble nuclear protein during extract preparation, although we also can’t rule out the possibility that this specific Esrp1 isoform shuttles between the nucleus and the cytoplasm. To determine how these alternative 5′ splice sites are used in endogenous *Esrp1* pre-mRNAs, we designed primers flanking both 5′ splice sites and confirmed alternative splicing of these 5′ splice sites across a panel of human, mouse, and rat cell lines with some variation in ratios between the splice variants (Supplementary Fig. S1). To further determine how both isoforms are produced at protein level endogenously, we obtained nuclear and cytoplasmic fractions for H358 cells. The Western blot results supported the presence of Esrp1 in both nucleus and cytoplasm, although the differences in size of isoforms with or without CKLP cannot be resolved by our Western analysis (Supplementary Fig. S1).

**Table 1:** Quantification of cells from each transfection on the subcellular localization of target protein.

	Total	N > C	N = C	N < C	Conclusion
**Full length Esrp1, related to Fig.** 1c					
2A + CKLP	34	34	0	0	N
2A − CKLP	62	1	4	57	C
NA + CKLP	47	45	2	0	N
NA − CKLP	21	0	1	20	C
**Chicken pyruvate kinase reporter, related to Fig.** 2c					
EV	34	0	0	34	C
SV 40 NLS	38	35	3	0	N
G rich	68	2	1	65	C
TP rich	89	0	6	83	C
15-mer + CKLP	35	23	12	0	N
15-mer − CKLP	88	0	3	85	C
**Chicken pyruvate kinase reporter, related to Fig.** 3					
CKLP- > AAAA	108	3	4	101	C
G1A	23	15	7	1	N
L2A	35	5	23	7	M
L2V	63	6	36	21	M
S3A	166	36	105	25	M
S3D	102	73	25	5	N
P4A	39	0	1	38	C
P5A	39	0	0	39	C
P6A	54	0	1	53	C
C7A	54	31	13	10	N
K8A	53	1	1	51	C
K8R	47	0	2	45	C
L9A	32	0	1	31	C
L9V	37	23	12	2	N
P10A	170	20	76	74	M
C11A	69	0	0	69	C
L12A	76	0	4	72	C
S13A	80	7	56	17	M
S13D	50	4	35	11	M
P14A	90	34	46	10	M
P15A	45	0	31	12	M
**Subcellular localization of fusilli, related to Fig.** 4					
Fusilli D FL	41	41	0	0	N
Fusilli G FL	39	0	0	39	C
Fusilli D ΔC	46	0	3	43	C
Fusilli D T1	44	44	0	0	N
Fusilli D T2	46	0	0	46	C
Fusilli D T4	49	0	5	44	C
Peptide 1	111	111	0	0	N
Peptide 2	91	0	1	90	C
Peptide 3	61	61	0	0	N

N > C represents predominantly nuclear; N = C represents similar presence in the nucleus and cytoplasm; N < C represents predominantly cytoplasmic.

### Determination of the Esrp1 sequence element that confers nuclear localization

**Figure 2:**
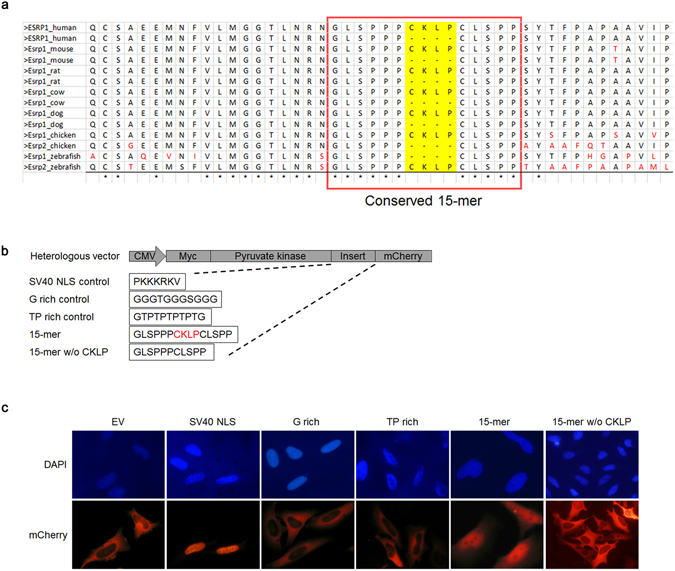
Determination of the Esrp1 sequence element that confers nuclear localization. (**a**) Alignment of Esrp1 and Esrp2 sequences from different species revealed high conservation around the CKLP region. The CKLP peptide is highlighted in yellow, amino acids that are conserved in all species are indicated by an asterisk, and amino acids that are deviated from the conservation are highlighted in red. The region of the proteins represented corresponds to amino acids 522–567 of the 681 amino acids of the human reference protein. The 15 conserved amino acids tested for nuclear localization are boxed. (**b**) Schematic of the reporter and peptide sequences used for transfection and microscopy in (**c**). (**c**) Representative images of HeLa cells (20x) transfected with different reporter vectors depicted in (**b**), from which we concluded that the fifteen amino acid peptide “GLSPPPCKLPCLSPP” is sufficient for nuclear localization. EV indicated the empty reporter construct.

To further characterize the peptide sequence that is necessary and sufficient for nuclear localization, we aligned Esrp1 and Esrp2 sequences from different species and focused on peptide sequences around the CKLP region to identify conserved amino acids (Fig. 2a). Importantly, this analysis also revealed that the expression of isoforms that do or do not contain CKLP is highly conserved across vertebrate species. Notably, due to the incomplete annotation of genomes, we were not able to identify both isoforms for some species. Based on the sequence conservation in the alignment, we selected several peptides around the CKLP region, and cloned them into a well-defined cDNA reporter that has previously been used to identify and characterize NLSs^18, 19^. This vector encodes chicken pyruvate kinase (18–443 aa), a cytoplasmic protein and we further modified this reporter by adding a fluorescent tag (mCherry) to the C-terminus. We then inserted sequences encoding these peptides from Esrp1 that included CKLP into the reporter and tested whether they could translocate chicken pyruvate kinase into the nucleus of HeLa cells (Fig. 2a,b). The SV40 NLS was used as a positive control and two unrelated peptides (G-rich and TP-rich sequences) were used as negative controls. In conclusion, we identified a fifteen-amino-acid stretch “GLSPPPCKLPCLSPP” that is sufficient for the nuclear localization while the same peptide excluding CKLP showed a cytoplasmic localization (Fig. 2c). The fact that this sequence confers nuclear localization both in the context of the Esrp1 protein and a widely used NLS reporter construct makes it most likely that it does function as an NLS, but cannot rule out that at least some of this function could be accounted for by promoting nuclear retention. We therefore will subsequently refer to this element as a putative nuclear localization signal (pNLS).

### Determination of key residues in the Esrp1 pNLS that are required for the nuclear localization

**Figure 3:**
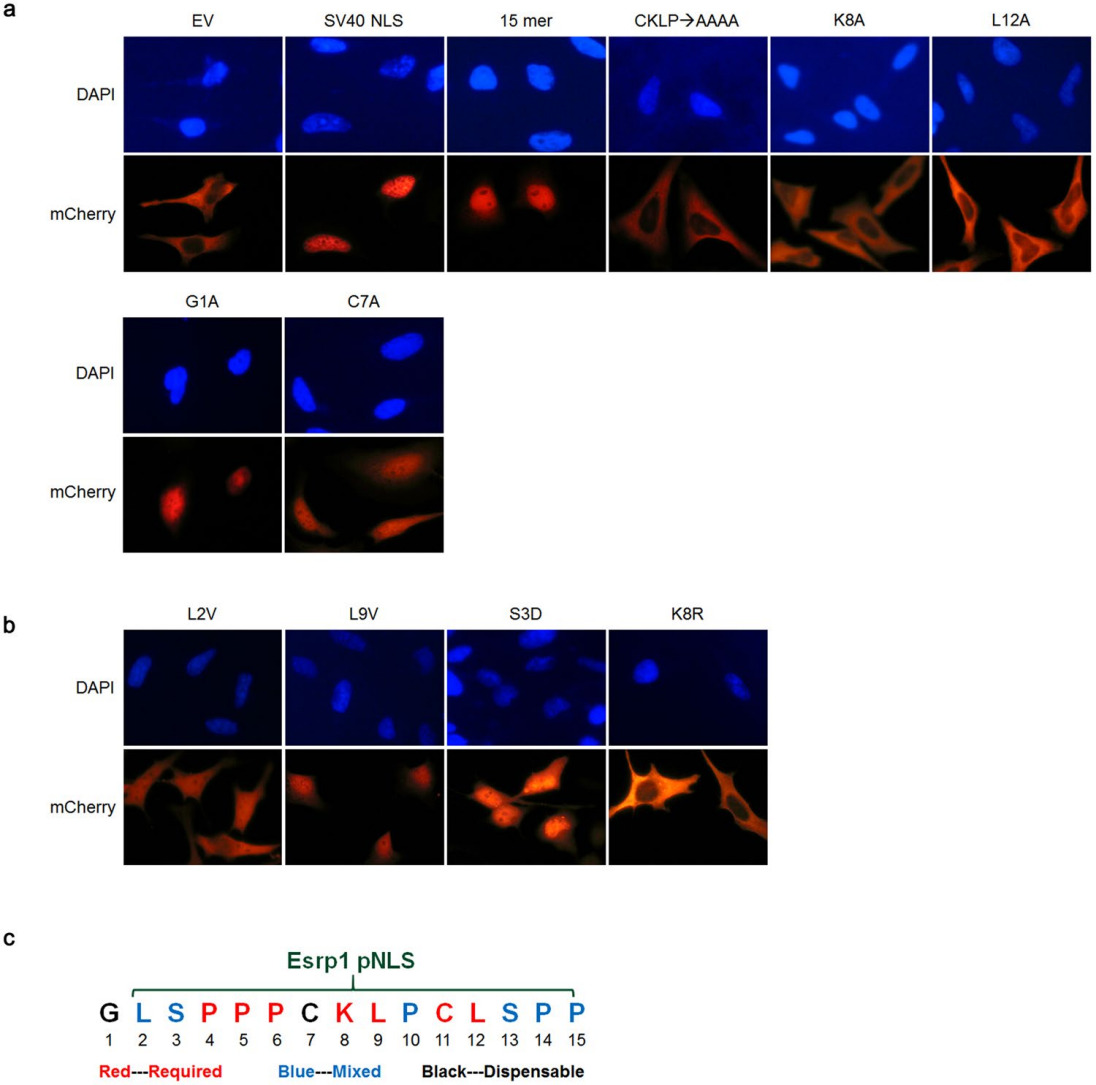
Determination of key residues in the Esrp1 pNLS that are required for nuclear localization. (**a**) Representative images of HeLa cells (20x) transfected with reporter vectors containing the control SV40 NLS and the 15 amino acid Esrp1 sequence as well as representative examples of amino acid substitutions with alanine that abolished (e.g. K8A and L12A) or preserved (e.g. G1A and C7A) nuclear localization. The numbers indicate amino acid positions within the 15 amino acid Esrp1 sequence as shown in (**c**). (**b**) Examples of other amino acid substitutions. (**c**) Summary of key residues in the fifteen amino acid Esrp1 sequence based on alanine substitution. Since the first Glycine is not required for nuclear localization, “LSPPPCKLPCLSPP” is the minimal Esrp1 sequence required for nuclear localization.

After identification of a 15-amino acid peptide sequence sufficient for nuclear localization, we sought to further define the key amino acids within the element that are required for nuclear import. We therefore introduced single amino acid mutations at each position in the Esrp1 pNLS and examined their effects on subcellular localization using the chicken pyruvate kinase vector. Mutation of CKLP to AAAA completely abolished the nuclear localization (Fig. 3a). Additionally, we identified seven amino acids for which single alanine substitution led to nearly complete cytoplasmic localization, six amino acids that displayed a mixed cytoplasmic and nuclear localization after alanine substitution, and two that remained nuclear after mutation (Fig. 3a). Since Leucine and Valine are both nonpolar amino acids and share a similar structure, we also mutated L to V at 2^nd^ and 9^th^ position. Interestingly, L2A and L2V both showed a mixed phenotype, in contrast, while L9A completely abolished nuclear localization, L9V remained nuclear, suggesting L9V preserves nuclear localization (Fig. 3b). It has been reported that phosphorylation within NLSs can positively or negatively affect nuclear import^20, 21^. The Serine residue at the third position in the Esrp1 pNLS is a known phosphorylation site that is also conserved in human^22–25^. Therefore, we generated a mutation which mimics the phosphorylated status of serine residue. While S3A exhibited a mixed cytoplasmic and nuclear localization, S3D restored nearly complete nuclear import, suggesting that phosphorylation of Serine at this position may play a role in facilitating the nuclear localization of Esrp1 (Fig. 3b). It will be of interest in future studies to determine conditions under which phosphorylation at this position might regulate Esrp1 localization. Since “Glycine” at the first position is not required for the nuclear localization, the minimal Esrp1 sequence conferring nuclear localizations is “LSPPPCKLPCLSPP” (Fig. 3c).

### Fusilli, the *D*. *Melanogaster* ortholog of Esrp1 also expresses both nuclear and cytoplasmic isoforms as a result of alternative splicing

**Figure 4:**
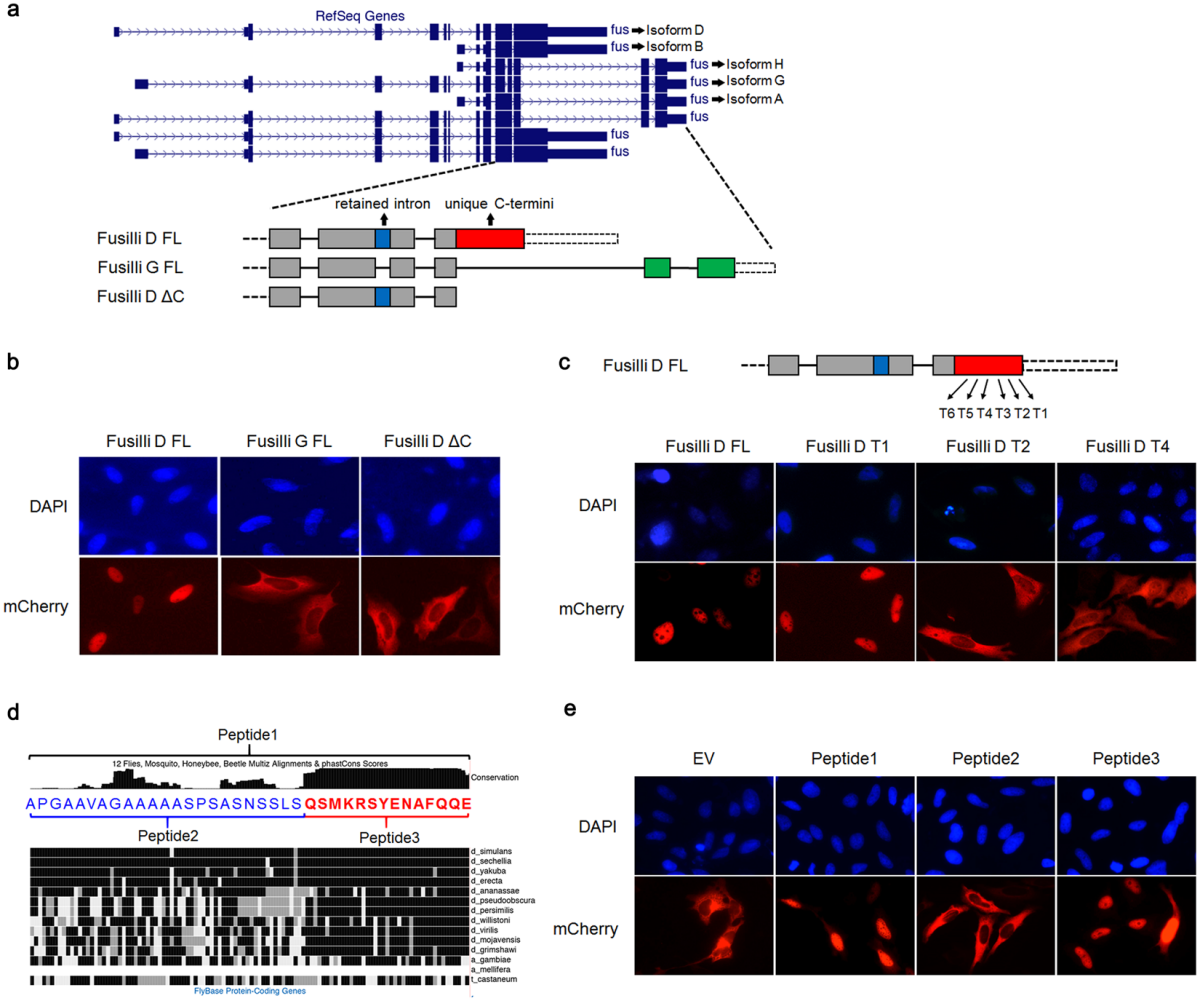
Fusilli expresses both nuclear and cytoplasmic isoforms as a result of alternative splicing. (**a**) A screenshot of the genome browser view with all fusilli isoforms and schematic of isoform D and G. fusilli D ΔC represents the C-terminal truncation of isoform D. Gray boxes indicate constitutive exons. Blue box indicates the retained intron; red and green boxes represent unique C-termini. (**b**) Representative images of HeLa cells (20x) transfected with expression vectors of different fusilli isoforms as mCherry fusion depicted in (**a**), from which we concluded that the C-terminus of isoform D is required for nuclear localization. (**c**) Representative images of HeLa cells (20x) transfected with expression vectors of multiple truncations from the C-terminus of isoform D, from which we concluded that the sequence required for nuclear localization of fusilli resides in the region between T1 and T2. (**d**) Alignment of the 37 amino acid peptide between T1 and T2 (peptide 1) of fusilli from different species revealed a highly conserved fourteen amino acid peptide sequence (peptide 3). (**e**) Representative images of HeLa cells (20x) transfected with different reporter vectors confirmed that peptide 3 is sufficient for nuclear localization.

The conservation of the alternative splicing event leading to both nuclear and cytoplasmic isoforms among Esrp1 orthologs in vertebrates suggested functional roles for both protein isoforms in post-transcriptional regulation. We sought to further investigate the degree of conservation for the expression of nuclear and cytoplasmic Esrp1 orthologs through alternative splicing by studying the subcellular localization of different isoforms of fusilli, the Esrp1 ortholog in *D*. *Melanogaster*. We previously showed that fusilli can regulate alternative splicing when ectopically expressed in mammalian cells, strongly suggesting that it has a conserved role as a splicing factor^12^. We noted that the fusilli gene has several alternative promoters and splicing events that generate multiple protein isoforms (Fig. 4a). Three splicing mRNA variants (A, B and H) result from a distal promoter and lack the majority of the first highly conserved RRM and therefore were not studied further. All other transcript variants encoding longer isoforms that contain all three RRMs are derived from alternative splicing, including a 93 nt retained intron within exon 9 and an alternative 5′ splice site coupled to alternative polyadenylation (APA5) that leads to distinct C-termini (Fig. 4a). We were able to generate cDNAs encoding isoform D that contains the retained intron as well as isoform G that utilizes the alternative 5′ splice site (Fig. 4a). Transfection of cDNAs encoding both isoforms as mCherry fusion proteins into HeLa cells showed that isoform D was nuclear while isoform G was predominantly cytoplasmic (Fig. 4b). To determine whether a presumed NLS in isoform D was present within the region encoded by the retained intron or the C-terminus, we made a truncated form of isoform D lacking the region encoded by the distinct C-terminus and determined that it was cytoplasmic, similar to isoform G. We therefore concluded that the sequence conferring nuclear localization resides in the C-terminus of isoform D, not the retained intron (Fig. 4b). To further pinpoint this sequence, we generated multiple truncations from the C-terminus of isoform D and tested their subcellular localization (Fig. 4c). While truncation 1 preserved nuclear localization, all subsequent truncations generated isoforms that were cytoplasmic localized. Therefore, we narrowed down the peptide to the region between the end of truncation 2 and truncation 1, which is a 37 amino acid fragment (peptide 1). To guide our determination of the minimal peptide sequence that is sufficient for the nuclear localization, we aligned this peptide sequences in 12 different fly species as well as other insects and noted the last 14 amino acids (peptide 3) are highly conserved compared to the first 23 amino acids (peptide 2) (Fig. 4d). Using the previously described chicken pyruvate kinase reporter, we determined that peptide 3 “QSMKRSYENAFQQE” is sufficient for the nuclear localization, thereby accounting for nuclear localization of isoforms that contain this peptide in fusilli (Fig. 4e). These observations thus indicate that while the sequences used to derive both nuclear and cytoplasmic isoforms of fusilli differ from that in vertebrate Esrp1, there has been functional conservation of a mechanism to express both isoforms via alternative splicing, suggesting biological significance over a long period of evolution.

## Discussion

It has become increasingly appreciated that many RBPs play multiple roles in mRNA-processing^1^. The subcellular localization of RBPs is one of many mechanisms that affect their regulatory functions. Our study adds Esrp1 and its orthologs to the list of RBPs that are known to expand their potential post-transcriptional regulatory functions through AS to generate both nuclear and cytoplasmic isoforms. The conservation in the fruit fly ortholog fusilli strongly suggests that there is an important biological function for both isoforms. While the alternative 5′ splice site only leads to a difference by four amino acids (CKLP) among isoforms, the functional consequences can be substantial. A well-known example, the Wilm’s Tumor (WT1) gene produces two protein isoforms that differ by only three amino acids (lysine-threonine-serine, or KTS), WT1 + KTS and WT1 − KTS, which are encoded by transcript variants that are alternatively spliced through the usage of two adjacent 5′ splice sites in the pre-mRNA^26^. While the WT1 − KTS is a transcription factor that binds to DNA and act as either transcriptional activator or repressor, the WT1 + KTS isoforms has been suggested to bind to RNA and regulate splicing or other post-transcriptional processes^27–30^. Since the 5′ splice sites in *Esrp1* that lead to +CKLP and −CKLP isoforms share the same 5′ splice site consensus sequence (CAT|GTAATG) and are separated by only 3 nucleotides, we initially surmised that the choice of either site during splicing was stochastic. However, we did note some differences in the ratios of nuclear and cytoplasmic isoforms in different cell lines that express Esrp1, suggesting the possibility that this splicing event may be regulated in different epithelial cell populations or at different stages of development. Regulation of such alternative splicing events can be fundamental for development. For example, there is a decrease in the inclusion of exon 5 in MBNL1 during cardiac morphogenesis^31^. Exon 5 is functionally important in regulating the subcellular localization as well as function of MNBL1. Specifically, isoforms containing exon 5 are predominantly nuclear while the loss of exon 5 leads to a cytoplasmic localization. Therefore, the nuclear localization of MBNL1 in the early stage of heart development is required for alternative splicing regulation and the loss of exon 5 is essential for the fetal-to-adult transitions of alternative splicing. Moreover, the alternative splicing of exon 19 in Rbfox1 is also regulated by nuclear Rbfox1 upon neuronal depolarization which leads to a switch from cytoplasmic to nuclear isoform^32^. While we have extensively characterized the role of Esrp1 in alternative splicing, the function of cytoplasmic Esrp1 requires further investigation. One study identified conserved Esrp binding motifs enriched in 3′UTRs and proposed a role for Esrp1 in the regulation of mRNA stability based on the correlation between the expression levels of Esrp1 and mRNAs with Esrp1 binding motifs in their 3′ UTRs among different tissues^33^. However, direct experimental evidence to support a function for Esrp1 as an mRNA stability regulator in the cytoplasm is required. On the other hand, another study provided evidence for a role of Esrp1 in negatively regulating translation of several pluripotency genes^17^. To broadly identify the direct mRNA targets and binding sites in the cytoplasm for Esrp1 *in vivo*, experiments such as RIP-Seq and/or CLIP-Seq in the cytoplasmic fraction will provide valuable information. Experiments such as metabolic labeling of nascent mRNA by pulsed nucleotide analogs followed by RNA-Seq and ribosome profiling will further define the roles for cytoplasmic Esrp1 in mRNA stability, translation or mRNA localization^34^.

A nuclear localization signal (NLS) is a short stretch of amino acids that mediates the transport of proteins into the nucleus^35^. The best characterized “classical NLS” (cNLS) consists of either one (monopartite) or two (bipartite) stretches of basic amino acids, for examples the well-known SV40 large antigen NLS and the nucleoplasmin NLS^18, 36^. Proteins containing a cNLS are translocated by the importin-α/β heterodimer. Importin-α (Impα; also known as Karyopherin-α) is a protein adaptor that directly binds to the cNLS and importin-β (Impβ; also known as Karyopherin-β1), which is associated with nuclear pore complex (NPC) to facilitate the nuclear import^35^. The other well-characterized NLS is the “PY-NLS”. While the sequences for PY-NLSs show limited similarity, they usually consist of a loose N-terminal hydrophobic or basic motif and a C-terminal RX_2–5_PY motif ^37–39^. Proteins containing a PY-NLS, for example HnRNP A1, are imported by Karyopherin-β2 (Kapβ2)^40, 41^. However, many NLSs have been identified with highly diverse sequences that do not belong to either class^42^. In addition, only a limited number of transport cargos are known for other importins^39^. The putative NLS sequences identified in this study for both Esrp1 and fusilli don’t conform to any of the well-characterized NLS consensus sequences and potentially represent a novel class of NLS. It will also be of interest to further confirm its function as an NLS and determine whether it represents a larger class of NLS motifs and to define the import pathway that it uses to translocate to the nucleus to regulate splicing.

## Methods

### Plasmids

The pIBX-C-FF(B) expression plasmid for expression of FLAG tagged proteins was described previously^12^. We PCR amplified the cDNA sequence for mCherry and inserted it into the vector using NheI and NsiI to make the pIBX-C-FF(B)-mCherry vector for expression of mCherry fucion proteins. We then PCR amplified the cDNA sequences for different isoforms of mouse Esrp1 and cloned them into the pIBX-C-FF(B)-mCherry vector using EcoRV and NotI sites to make the expression vectors. pCMV-myc-PK vector was described previously^19^. PCR amplified coding sequence for EGFP was inserted into NheI and NsiI digested pCMV-myc-PK to drive pCMV-myc-PK-EGFP. We then PCR amplified the coding sequence for 2x FLAG tag followed by SV40 NLS and cloned it downstream of pyruvate kinase coding sequence and upstream of EGFP coding sequence to drive pCMV-myc-PK-FF-NLS-EGFP using Not I and NheI sites. We replaced the coding sequence for EGFP with that for mCherry to generate pCMV-myc-PK-FF-NLS-mCherry. We digested the vector with NotI and NheI in order to clone in other sequences. To determine the minimum peptide sequences sufficient for Esrp1 nuclear localization and to identify key residues in the Esrp1 pNLS, for the fifteen amino acid peptide and all tested mutants, the corresponding sense and antisense oligoes were annealed and ligated into the cut reporter vector. pIBX-C-FF(B)-fusilli D has been described before^12^. We cloned in mCherry coding sequence into NheI and NsiI digested pIBX-C-FF(B)-fusilli D to drive pIBX-C-FF(B)-fusilli D-mCherry. A series of reverse primer in the C-terminus of fusilli D and the universal forward T7 primer were used to make all the C-terminal truncation proteins. fusilli G was made by gene synthesis. To determine the minimum peptide sequences sufficient for fusilli nuclear localization, for peptide 1, 2 and 3, the corresponding sense and antisense oligoes were annealed and ligated into the cut reporter vector. Primers used for cloning are summarized in Supplementary Table S1. The pTet-On advanced vector was purchased from Clontech (631069). We cloned the rtTA-Advanced cassette into the pIBX vector described previously using EcoRI and BamHI sites to make the pIBX-Tet-On vector^12^. The pTRE-tight vector was purchased from Clontech (631059). To enable selection, we cloned the TRE-CMVmin element into the pcDNA3 vector to derive pcDNA3-TRE-CMVmin. A subsequent construct, pcDNA-TRE-CMVmini-C-FF(B)-mCherry was derived by inserting a sequence encoding a 2X FLAG tag followed by the coding sequence for mCherry. The coding sequence for Esrp1 2A + CKLP or Esrp1 2A − CKLP was subsequently inserted upstream of the sequences encoding the FLAG tag and mCherry to derive pcDNA3-TRE-CMVmin-C- FF(B)- mCherry-Esrp1 2A + CKLP or 2A − CKLP. Complete annotated sequence files and maps for all vectors are available on request.

### RNA extraction and RT-PCR

Total RNA was extracted using TRIzol (15596018, Life technologies). Reverse transcription was performed as described previously^12^. Primers used to detect the nuclear and cytoplasmic isoforms are in Supplementary Table S1.

### Cell culture and transfection

HeLa cells were maintained in DMEM medium with 10% FBS (SH30071.03, GE). Human non-small cell lung cancer cell line H358 (obtained from the American Type Culture Collection) were maintained in RPMI1640 with 10% FBS. To make the tet-on inducible H358 stable clone that express either Esrp1 2A + CKLP or Esrp1 2A-CKLP: First, H358 cells were transfected with the pIBX-Tet-On plasmid using Lipofectamine® 2000 (11668027, Life technologies) according the manufacturers’ protocols, selected in 10 ug/ml Blasticidin for two weeks, and single cell derived clones were obtained using serial dilution in 96 well plates. Second, pcDNA3-TRE-CMVmin-C- FF(B)- mCherry-Esrp1 2A + CKLP or 2A−CKLP was transfected into the H358 Tet-On clone using Lipofectamine® 2000, selected in 500 ug/ml G418 (10131035, Life technologies) and single cell derived clones were obtained by serial dilution. To induce the expression of Esrp1 protein isoforms, the Esrp1 2A + CKLP and 2A−CKLP stable clones were treated with 25 ng/ml or 5 ng/ml doxycycline for 16 hours respectively. For transfection, HeLa cells were seeded on coverslips in 6-well plates at 200,000 cells per well and incubated overnight. Cells were transfected using Lipofectamine® 2000 according the manufacturers’ protocols. 2 ug of plasmids were used for each transfection.

### Antibodies and Western blotting

Total cell extracts were harvested in RIPA buffer with protease inhibitor cocktail, PMSF and sodium orthovanadate (sc-24948, Santa Cruz). Cytoplasmic fractions were obtained using buffer A (10 mM HEPES pH 7.9, 1.5 mMgCl_2_, 1 mM DTT) with 0.2% NP-40 and protease inhibitor cocktail; nuclear fractions were obtained using 1x PXL buffer (1x PBS without Mg^2+^/Ca^2+^, 0.1% SDS, 0.5% NP-40) with protease inhibitor cocktail. Immunoblotting was performed as described^12^. Antibodies used are as follows: ESRP1 (27H12, mouse, 1:200)^13^; FLAG (F1804, Sigma, mouse, 1:5000); β-tubulin (T8328, Sigma, mouse, 1:5000); mouse monoclonal U2AF65 (kindly provided by Juan Valcarcel, 1:5000). Secondary antibody was purchased from GE Healthcare Life Sciences (sheep anti-Mouse IgG NA931 from 1:2000 to 1:10000).

### Microscopy

24 hours post transfection, the coverslip containing transfected cells was washed once with cold 1xPBS, the cells were then fixed with acetone for three minutes and washed with cold 1XPBS for four times. The coverslips were mounted to a clear glass slide using mounting media with DAPI staining (P36931, Thermo Scientific). For each transfection, at least three randomly chosen microscopic fields were imaged. At least 20 cells were qualitatively measured for the subcellular localization as follows: N > C represents predominantly nuclear; N = C represents similar presence in the nucleus and cytoplasm; N < C represents predominantly cytoplasmic. These quantifications are summarized in Table 1.

## Electronic supplementary material

Supplementary Information

## References

[1] Keene JD (2007). RNA regulons: coordination of post-transcriptional events. Nat Rev Genet.

[2] Mukherjee N (2011). Integrative regulatory mapping indicates that the RNA-binding protein HuR couples pre-mRNA processing and mRNA stability. Mol Cell.

[3] Sawicka K, Bushell M, Spriggs KA, Willis AE (2008). Polypyrimidine-tract-binding protein: a multifunctional RNA-binding protein. Biochem Soc Trans.

[4] Vanharanta, S. *et al*. Loss of the multifunctional RNA-binding protein RBM47 as a source of selectable metastatic traits in breast cancer. *Elife* **3**, doi:10.7554/eLife.02734 (2014). 10.7554/eLife.02734 PMC4073284 24898756

[5] Simone LE, Keene JD (2013). Mechanisms coordinating ELAV/Hu mRNA regulons. Current Opinion in Genetics & Development.

[6] Hall MP (2013). Quaking and PTB control overlapping splicing regulatory networks during muscle cell differentiation. RNA.

[7] Saccomanno L (1999). The STAR protein QKI-6 is a translational repressor. Proceedings of the National Academy of Sciences.

[8] Doukhanine E, Gavino C, Haines JD, Almazan G, Richard S (2010). The QKI-6 RNA Binding Protein Regulates Actin-interacting Protein-1 mRNA Stability during Oligodendrocyte Differentiation. Molecular Biology of the Cell.

[9] Li Z, Zhang Y, Li D, Feng Y (2000). Destabilization and mislocalization of myelin basic protein mRNAs in quaking dysmyelination lacking the QKI RNA-binding proteins. J Neurosci.

[10] Lee JA (2016). Cytoplasmic Rbfox1 Regulates the Expression of Synaptic and Autism-Related Genes. Neuron.

[11] Yang Y (2016). Determination of a Comprehensive Alternative Splicing Regulatory Network and Combinatorial Regulation by Key Factors during the Epithelial-to-Mesenchymal Transition. Molecular and Cellular Biology.

[12] Warzecha CC, Sato TK, Nabet B, Hogenesch JB, Carstens RP (2009). ESRP1 and ESRP2 are epithelial cell-type-specific regulators of FGFR2 splicing. Mol Cell.

[13] Warzecha CC (2010). An ESRP-regulated splicing programme is abrogated during the epithelial-mesenchymal transition. EMBO J.

[14] Shapiro IM (2011). An EMT-driven alternative splicing program occurs in human breast cancer and modulates cellular phenotype. PLoS Genet.

[15] Bebee, T. W. *et al*. The splicing regulators Esrp1 and Esrp2 direct an epithelial splicing program essential for mammalian development. *Elife* **4**, doi:10.7554/eLife.08954 (2015). 10.7554/eLife.08954 PMC4566030 26371508

[16] Barberan-Soler S, Zahler AM (2008). Alternative splicing regulation during C. elegans development: splicing factors as regulated targets. PLoS Genet.

[17] Fagoonee S (2013). The RNA Binding Protein ESRP1 Fine-Tunes the Expression of Pluripotency-Related Factors in Mouse Embryonic Stem Cells. Plos One.

[18] Kalderon D, Roberts BL, Richardson WD, Smith AE (1984). A short amino acid sequence able to specify nuclear location. Cell.

[19] Siomi H, Dreyfuss G (1995). A nuclear localization domain in the hnRNP A1 protein. J Cell Biol.

[20] Kaffman A, O’Shea EK (1999). Regulation of nuclear localization: a key to a door. Annu Rev Cell Dev Biol.

[21] Jans, D. A., Xiao, C.-Y. & Lam, M. H. C. Nuclear targeting signal recognition: a key control point in nuclear transport? *BioEssays* **22**, 532–544, doi:10.1002/(SICI)1521-1878 (2000). 10.1002/(SICI)1521-1878(200006)22:6<532::AID-BIES6>3.0.CO;2-O 10842307

[22] Van Hoof D (2009). Phosphorylation Dynamics during Early Differentiation of Human Embryonic Stem Cells. Cell Stem Cell.

[23] Klammer M (2012). Phosphosignature Predicts Dasatinib Response in Non-small Cell Lung Cancer. Molecular & Cellular Proteomics.

[24] Mertins P (2014). Ischemia in Tumors Induces Early and Sustained Phosphorylation Changes in Stress Kinase Pathways but Does Not Affect Global Protein Levels. Molecular & Cellular Proteomics.

[25] Rigbolt KTG (2011). System-Wide Temporal Characterization of the Proteome and Phosphoproteome of Human Embryonic Stem Cell Differentiation. Science Signaling.

[26] Haber DA (1991). Alternative splicing and genomic structure of the Wilms tumor gene WT1. Proc Natl Acad Sci USA.

[27] Larsson SH (1995). Subnuclear localization of WT1 in splicing or transcription factor domains is regulated by alternative splicing. Cell.

[28] Huff V (2011). Wilms’ tumours: about tumour suppressor genes, an oncogene and a chameleon gene. Nat Rev Cancer.

[29] Markus MA (2006). WT1 interacts with the splicing protein RBM4 and regulates its ability to modulate alternative splicing *in vivo*. Experimental Cell Research.

[30] Hammes A (2001). Two Splice Variants of the Wilms’ Tumor 1 Gene Have Distinct Functions during Sex Determination and Nephron Formation. Cell.

[31] Terenzi F, Ladd AN (2010). Conserved developmental alternative splicing of muscleblind-like (MBNL) transcripts regulates MBNL localization and activity. RNA Biol.

[32] Lee JA, Tang ZZ, Black DL (2009). An inducible change in Fox-1/A2BP1 splicing modulates the alternative splicing of downstream neuronal target exons. Genes Dev.

[33] Ray, D. *et al*. A compendium of RNA-binding motifs for decoding gene regulation. *Nature* **499**, 172–177, doi:10.1038/nature12311http://www.nature.com/nature/journal/v499/n7457/abs/nature12311.html#supplementary-information (2013). 10.1038/nature12311 PMC3929597 23846655

[34] Rabani, M. *et al*. Metabolic labeling of RNA uncovers principles of RNA production and degradation dynamics in mammalian cells. *Nat Biotech* **29**, 436–442, doi:10.1038/nbt.1861http://www.nature.com/nbt/journal/v29/n5/abs/nbt.1861.html#supplementary-information (2011). 10.1038/nbt.1861 PMC3114636 21516085

[35] Lange A (2007). Classical Nuclear Localization Signals: Definition, Function, and Interaction with Importin α. Journal of Biological Chemistry.

[36] Robbins J, Dilwortht SM, Laskey RA, Dingwall C (1991). Two interdependent basic domains in nucleoplasmin nuclear targeting sequence: Identification of a class of bipartite nuclear targeting sequence. Cell.

[37] Lee BJ (2006). Rules for Nuclear Localization Sequence Recognition by Karyopherinβ2. Cell.

[38] Süel KE, Gu H, Chook YM (2008). Modular Organization and Combinatorial Energetics of Proline? Tyrosine Nuclear Localization Signals. PLoS Biol.

[39] Xu D, Farmer A, Chook YM (2010). Recognition of nuclear targeting signals by Karyopherin-β proteins. Current Opinion in Structural Biology.

[40] Bonifaci N, Moroianu J, Radu A, Blobel G (1997). Karyopherin β2 mediates nuclear import of a mRNA binding protein. Proceedings of the National Academy of Sciences.

[41] Pollard VW (1996). A Novel Receptor-Mediated Nuclear Protein Import Pathway. Cell.

[42] Soniat M, Chook, Yuh M (2015). Nuclear localization signals for four distinct karyopherin-β nuclear import systems. Biochemical Journal.

