# Supplementary material for: Alternative splicing regulates distinct subcellular localization of Epithelial splicing regulatory protein 1 (Esrp1) isoforms
Source: Sci Rep. 2017 Jun 20;7:3848. doi: 10.1038/s41598-017-03180-3 (PMC5478600; doi:10.1038/s41598-017-03180-3)

Supplementary Information for

Alternative splicing regulates distinct subcellular localization of Epithelial splicing regulatory protein 1 (Esrp1) isoforms

Yueqin Yang[1, 2], Russ P. Carstens[1, 2 *]

Departments of Genetics[1] and Medicine[2], Perelman School of Medicine, University of Pennsylvania, Philadelphia, Pennsylvania, 19104, USA

* Corresponding author: Russ P. Carstens russcars@upenn.edu;

Supplementary Figure S1

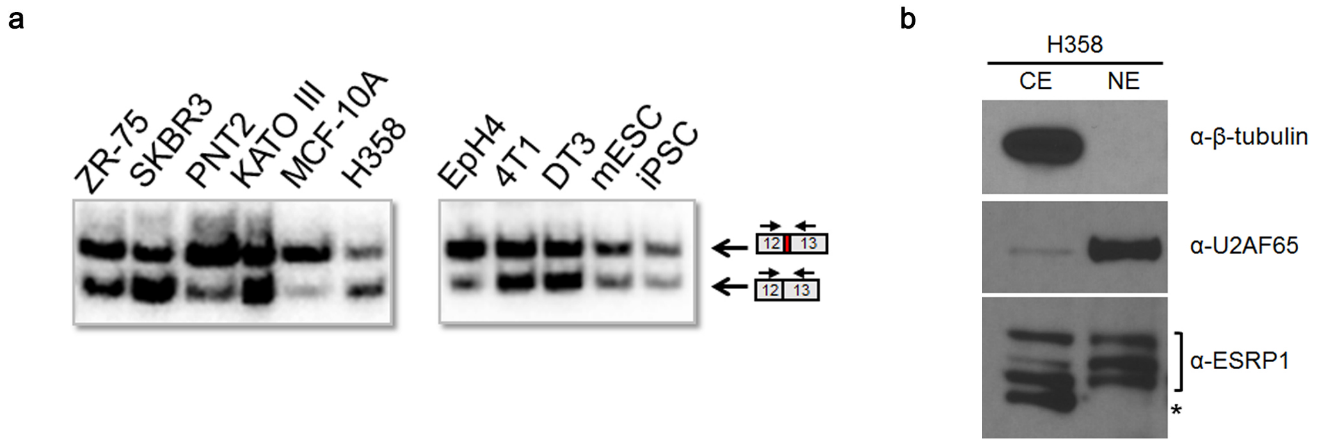

Supplementary Figure S1. Splice variants that contain or lack CKLP are present in a panel of cell lines that express Esrp1 and endogenous ESRP1 is present in both the nucleus and cytoplasm of H358 cells. (a) RT-PCR using primers flanking both 5’ splice sites across a panel of human, mouse and rat cell lines confirmed the expression of both splice variants in these cells. The top band represents splice variants using the distal 5’ splice site, the bottom band represents splice variants using the proximal 5’ splice site. The arrows indicate the locations of primers. Notably, the RNAs were obtained from cells grown at different states of confluence. ZR-75, human breast cancer cell line; SKBR3, human breast cancer cell line; PNT2, human prostate immortalized cell line; KATO III, human gastric cancer cell line; MCF-10A, human mammary gland epithelial cell line; H358, human lung cancer cell line; EpH4, mouse breast cancer cell line; 4T1, mouse mammary cancer cell line; DT3, rat prostate cancer cell line; mouse embryonic stem cell (mESC) line V6.5 and induced pluripotency stem cell (iPSC).

(b) Western blot of nuclear and cytoplasmic fractions from H358 cells supported the presence of endogenous ESRP1 proteins in both the nucleus and cytoplasm. β-tubulin was used as a cytoplasmic marker, and U2AF65 was used as a nuclear marker. An asterisk represents a non-specific cross-reacting band present in the cytoplasm that our previous studies have shown is not Esrp1.

Supplementary Table S1

Forward_humanESRP1 exon 12	GCACTTTAAATCGAAATGGCTTAT
Reverse_humanESRP1 exon 13	AGCAGGAGCTGGAAATGTGT
Forward_mouseEsrp1 exon 12	CAGCTGAGGAGATGAACTTTGTG
Reverse_mouseEsrp1 exon 13	GGGAATGTGTAGGAGGGAGGAGAC
Sense_15mer+CKLP	GGCCGCCGGCTTGTCCCCACCGCCATGTAAGTTACCATGCCTGTCTCCTCCCG
Antisense_15-mer+CKLP	CTAGCGGGAGGAGACAGGCATGGTAACTTACATGGCGGTGGGGACAAGCCGGC
Sense_15mer-CKLP	GGCCGCCGGCTTGTCCCCACCGCCATGCCTGTCTCCTCCCG
Antisense_15mer-CKLP	CTAGCGGGAGGAGACAGGCATGGCGGTGGGGACAAGCCGGC
Sense_CKLP-> AAAA	GGCCGCCGGCTTGTCCCCACCGCCAGCTGCGGCAGCATGCCTGTCTCCTCCCG
Antisense_CKLP-> AAAA	CTAGCGGGAGGAGACAGGCATGCTGCCGCAGCTGGCGGTGGGGACAAGCCGGC
Sense_G1A	GGC CGC CGC CTT GTC CCC ACC GCC ATG TAA GTT ACC ATG CCT GTC TCC TCC CG
Antisense_G1A	CTA GCG GGA GGA GAC AGG CAT GGT AAC TTA CAT GGC GGT GGG GAC AAG GCG GC
Sense_L2A	GGC CGC CGG CGC GTC CCC ACC GCC ATG TAA GTT ACC ATG CCT GTC TCC TCC CG
Antisense_L2A	CTA GCG GGA GGA GAC AGG CAT GGT AAC TTA CAT GGC GGT GGG GAC GCG CCG GC
Sense_L2V	GGC CGC CGG CGT GTC CCC ACC GCC ATG TAA GTT ACC ATG CCT GTC TCC TCC CG
Antisense_L2V	CTA GCG GGA GGA GAC AGG CAT GGT AAC TTA CAT GGC GGT GGG GAC ACG CCG GC
Sense_S3A	GGC CGC CGG CTT GGC CCC ACC GCC ATG TAA GTT ACC ATG CCT GTC TCC TCC CG
Antisense_S3A	CTA GCG GGA GGA GAC AGG CAT GGT AAC TTA CAT GGC GGT GGG GCC AAG CCG GC
Sense_S3D	GGC CGC CGG CTT GGA CCC ACC GCC ATG TAA GTT ACC ATG CCT GTC TCC TCC CG
Antisense_S3D	CTA GCG GGA GGA GAC AGG CAT GGT AAC TTA CAT GGC GGT GGG TCC AAG CCG GC
Sense_P4A	GGC CGC CGG CTT GTC CGC ACC GCC ATG TAA GTT ACC ATG CCT GTC TCC TCC CG
Antisense_P4A	CTA GCG GGA GGA GAC AGG CAT GGT AAC TTA CAT GGC GGT GCG GAC AAG CCG GC
Sense_P5A	GGC CGC CGG CTT GTC CCC AGC GCC ATG TAA GTT ACC ATG CCT GTC TCC TCC CG
Antisense_P5A	CTA GCG GGA GGA GAC AGG CAT GGT AAC TTA CAT GGC GCT GGG GAC AAG CCG GC
Sense_P6A	GGC CGC CGG CTT GTC CCC ACC GGC ATG TAA GTT ACC ATG CCT GTC TCC TCC CG
Antisense_P6A	CTA GCG GGA GGA GAC AGG CAT GGT AAC TTA CAT GCC GGT GGG GAC AAG CCG GC
Sense_C7A	GGC CGC CGG CTT GTC CCC ACC GCC AGC TAA GTT ACC ATG CCT GTC TCC TCC CG
Antisense_C7A	CTA GCG GGA GGA GAC AGG CAT GGT AAC TTA GCT GGC GGT GGG GAC AAG CCG GC
Sense_K8A	GGC CGC CGG CTT GTC CCC ACC GCC ATG TGC GTT ACC ATG CCT GTC TCC TCC CG
Antisense_K8A	CTA GCG GGA GGA GAC AGG CAT GGT AAC GCA CAT GGC GGT GGG GAC AAG CCG GC
Sense_K8R	GGC CGC CGG CTT GTC CCC ACC GCC ATG TAG GTT ACC ATG CCT GTC TCC TCC CG
Antisense_K8R	CTA GCG GGA GGA GAC AGG CAT GGT AAC CTA CAT GGC GGT GGG GAC AAG CCG GC
Sense_L9A	GGC CGC CGG CTT GTC CCC ACC GCC ATG TAA GGC ACC ATG CCT GTC TCC TCC CG
Antisense_L9A	CTA GCG GGA GGA GAC AGG CAT GGT GCC TTA CAT GGC GGT GGG GAC AAG CCG GC

Sense_L9V	GGC CGC CGG CTT GTC CCC ACC GCC ATG TAA GGT ACC ATG CCT GTC TCC TCC CG
Antisense_L9V	CTA GCG GGA GGA GAC AGG CAT GGT ACC TTA CAT GGC GGT GGG GAC AAG CCG GC
Sense_P10A	GGC CGC CGG CTT GTC CCC ACC GCC ATG TAA GTT AGC ATG CCT GTC TCC TCC CG
Antisense_P10A	CTA GCG GGA GGA GAC AGG CAT GCT AAC TTA CAT GGC GGT GGG GAC AAG CCG GC
Sense_C11A	GGC CGC CGG CTT GTC CCC ACC GCC ATG TAA GTT ACC AGC CCT GTC TCC TCC CG
Antisense_C11A	CTA GCG GGA GGA GAC AGG GCT GGT AAC TTA CAT GGC GGT GGG GAC AAG CCG GC
Sense_L12A	GGC CGC CGG CTT GTC CCC ACC GCC ATG TAA GTT ACC ATG CGC GTC TCC TCC CG
Antisense_L12A	CTA GCG GGA GGA GAC GCG CAT GGT AAC TTA CAT GGC GGT GGG GAC AAG CCG GC
Sense_S13A	GGC CGC CGG CTT GTC CCC ACC GCC ATG TAA GTT ACC ATG CCT GGC TCC TCC CG
Antisense_S13A	CTA GCG GGA GGA GCC AGG CAT GGT AAC TTA CAT GGC GGT GGG GAC AAG CCG GC
Sense_S13D	GGC CGC CGG CTT GTC CCC ACC GCC ATG TAA GTT ACC ATG CCT GGA TCC TCC CG
Antisense_S13D	CTA GCG GGA GGA TCC AGG CAT GGT AAC TTA CAT GGC GGT GGG GAC AAG CCG GC
Sense_P14A	GGC CGC CGG CTT GTC CCC ACC GCC ATG TAA GTT ACC ATG CCT GTC TGC TCC CG
Antisense_P14A	CTA GCG GGA GCA GAC AGG CAT GGT AAC TTA CAT GGC GGT GGG GAC AAG CCG GC
Sense_P15A	GGC CGC CGG CTT GTC CCC ACC GCC ATG TAA GTT ACC ATG CCT GTC TCC TGC CG
Antisense_P15A	CTA GCG GCA GGA GAC AGG CAT GGT AAC TTA CAT GGC GGT GGG GAC AAG CCG GC
T7_forward	TAATACGACTCACTATAGGG
Fus_D exon 10 _Reverse	GGGCGGCCGCACCCGTAGTGGCGGCCAG
Fus_D trunc1_Reverse	GGG CGG CCG CCT CCT GCT GAA AGG CGT TCT C
Fus_D trunc2_Reverse	GGG CGG CCG CCA CTC CAA CGC CCG ATG CCG C
Fus_D trunc3_Reverse	GGG CGG CCG CTT GTT GCA ACA TGG CAC TGG C
Fus_D trunc4_Reverse	GGG CGG CCG CAG CGG CCA AGG CAG CAG CTG C
Fus_D trunc5_Reverse	GGG CGG CCG CGG CGT AAC CCT GAG GTG CCA A
Fus_D trunc6_Reverse	GGG CGG CCG CGT GGG CGG CGG CGG CTG CCG C
Sense_peptide 1 fusilli	GGCCGCCGCGCCAGGAGCAGCAGTTGCTGGAGCGGCAGCAGCCGCATCGCCTTCCGCCAGCA ACTCCTCGCTGAGTCAATCGATGAAGCGCTCGTACGAGAACGCCTTTCAGCAGGAGG
Antisense_peptide 1 fusilli	CTAGCCTCCTGCTGAAAGGCGTTCTCGTACGAGCGCTTCATCGATTGACTCAGCGAGGAGTTGC TGGCGGAAGGCGATGCGGCTGCTGCCGCTCCAGCAACTGCTGCTCCTGGCGCGGC
Sense_peptide 2 fusilli	GGCCGCCGCGCCAGGAGCAGCAGTTGCTGGAGCGGCAGCAGCCGCATCGCCTTCCGCCAGCA ACTCCTCGCTGAGTG
Antisense_peptide 2 fusilli	CTAGCACTCAGCGAGGAGTTGCTGGCGGAAGGCGATGCGGCTGCTGCCGCTCCAGCAACTGCT GCTCCTGGCGCGGC
Sense_peptide 3 fusilli	GGCCGCCCAATCGATGAAGCGCTCGTACGAGAACGCCTTTCAGCAGGAGG
Antisense_peptide 3 fusilli	CTAGCCTCCTGCTGAAAGGCGTTCTCGTACGAGCGCTTCATCGATTGGGC

Full length gel for Figure 1d

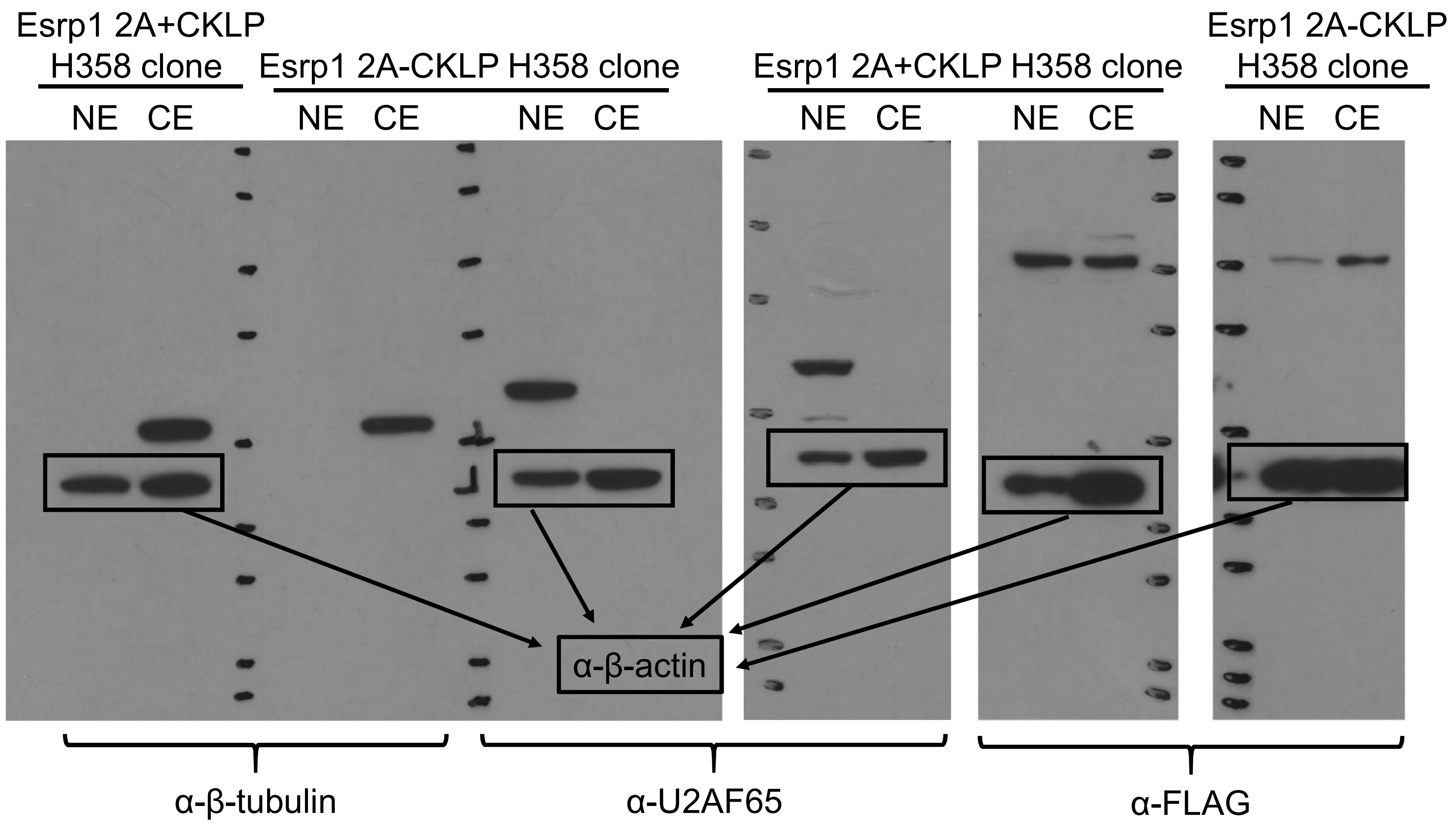

Full length gel for Supplementary Figure S1

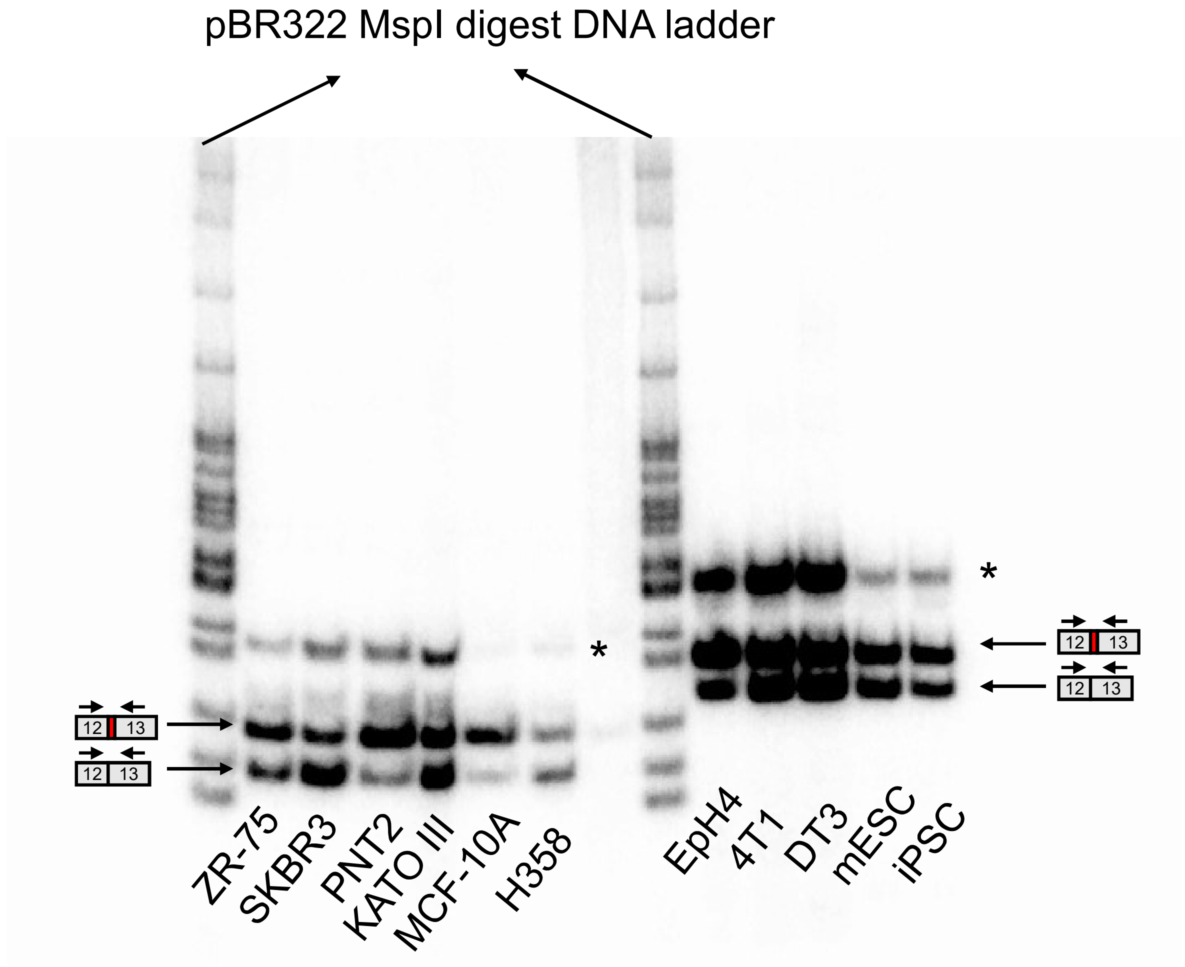

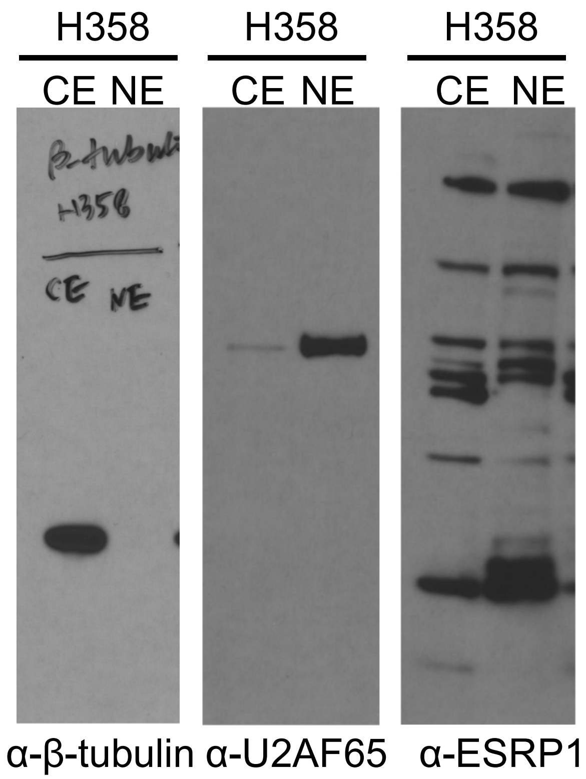

Supplement: Supplementary file 1 — Supplementary Information [file 41598_2017_3180_MOESM1_ESM.pdf]
